# EPIGENETIC MECHANISMS OF TISSUE AGING

**DOI:** 10.1093/geroni/igad104.0459

**Published:** 2023-12-21

**Authors:** Payel Sen

**Affiliations:** National Institute on Aging, National Institutes of Health, Baltimore, Maryland, United States

## Abstract

Epigenetic alterations are a hallmark of aging, affecting DNA and histone modifications, 3D genome structure, and gene expression. Our lab studies these changes in various tissues to understand their unique and shared characteristics. In this talk, I will focus on the skeletal muscle, which makes up ~40% of the body weight, contributes to ~30% of resting energy expenditure, and is affected by aging. Aging reduces muscle mass and strength, partly due to the reduced function of resident muscle stem cells (MuSCs). We used an integrative multi-omics approach to profile the transcriptome, epigenome, and 3D genome of MuSCs isolated from young, old, and geriatric mice and comprehensively understand why their regenerative potential diminishes with age. We found an elevated immune response and quiescence exit signature in MuSCs during aging and identified regulatory elements that contribute to these changes. By targeting these networks, we can potentially rejuvenate MuSC function. Overall, we show that the non-coding genome potentiates a functional drift in MuSCs, an otherwise non-immune cell to a more immune function.

